# Digital Health Strategies in Heart Failure: Effects of Telemedicine and Remote Monitoring on Clinical Outcomes—A Systematic Review and Meta-Analysis

**DOI:** 10.3390/jcm15103880

**Published:** 2026-05-18

**Authors:** Dan Alexandru Surducan, Madalin-Marius Margan, Dragos-Mihai Gavrilescu, Andrei Marginean, Diana-Maria Mateescu, Ioana Cotet, Cristina Tudoran, Roxana Folescu, Mihaela-Diana Popa, Sorin Ursoniu, Costela Serban, Adrian-Cosmin Ilie

**Affiliations:** 1 Department of Functional Sciences, Discipline of Public Health, Center for Translational Research and Systems Medicine, “Victor Babes” University of Medicine and Pharmacy Timisoara, Eftimie Murgu Square 2, 300041 Timisoara, Romania; surducan.dan@umft.ro (D.A.S.); margan.madalin@umft.ro (M.-M.M.); ilie.adrian@umft.ro (A.-C.I.); 2 Center for Translational Research and Systems Medicine, Faculty of Medicine, “Victor Babes” University of Medicine and Pharmacy Timisoara, Eftimie Murgu Square 2, 300041 Timisoara, Romania; 3 Department of Orthodontics, Dental District, Zăgazului 3, One Floreasca Vista, Sector 1, 014261 Bucharest, Romania; dr.gavrilescu@outlook.com; 4 Department of Surgery, Dr. Victor Popescu Emergency Military Hospital, Gheorghe Lazăr 9, 300080 Timisoara, Romania; andreivmarginean@yahoo.com; 5 Doctoral School, Department of General Medicine, “Victor Babes” University of Medicine and Pharmacy Timisoara, Eftimie Murgu Square 2, 300041 Timisoara, Romania; diana.mateescu@umft.ro (D.-M.M.); ioana.cotet@umft.ro (I.C.); 6 Centre of Molecular Research in Nephrology and Vascular Disease, “Victor Babes” University of Medicine and Pharmacy Timisoara, Eftimie Murgu Square 2, 300041 Timisoara, Romania; tudoran.cristina@umft.ro; 7 Department VII, Internal Medicine II, Discipline of Cardiology, “Victor Babes” University of Medicine and Pharmacy Timisoara, Eftimie Murgu Square 2, 300041 Timisoara, Romania; 8 County Emergency Hospital “Pius Brinzeu”, L. Rebreanu 156, 300723 Timisoara, Romania; 9 Department of Balneology, Medical Recovery and Rheumatology, Family Discipline, Center for Preventive Medicine, “Victor Babes” University of Medicine and Pharmacy Timisoara, Eftimie Murgu Square 2, 300041 Timisoara, Romania; folescu.roxana@umft.ro; 10 Department of Microbiology, “Victor Babes” University of Medicine and Pharmacy Timisoara, Eftimie Murgu Square 2, 300041 Timisoara, Romania; popa.mihaela@umft.ro

**Keywords:** heart failure, telemedicine, remote patient monitoring, haemodynamic-guided monitoring, structured remote patient management, non-invasive telemonitoring, all-cause mortality, hospitalization, digital health, meta-analysis

## Abstract

**Background/Objectives**: Telemedicine and remote patient monitoring have emerged as promising strategies to improve outcomes in heart failure (HF), but prior meta-analyses reported conflicting results, partly due to insufficient differentiation between intervention modalities. This systematic review and meta-analysis evaluated the impact of distinct telemedicine strategies on clinically relevant outcomes in HF. **Methods**: Conducted according to PRISMA 2020 and a prospectively registered PROSPERO protocol (CRD420261355507), this analysis included randomized controlled trials (RCTs) comparing telemedicine-based strategies—non-invasive telemonitoring, structured remote patient management (RPM), or haemodynamic-guided monitoring—against standard care, identified through searches of PubMed/MEDLINE, Embase, and CENTRAL (inception to 15 March 2026). Random-effects meta-analyses (DerSimonian–Laird) were performed, with predefined subgroup, sensitivity, and publication bias analyses. **Results**: Sixteen RCTs (*n* = 8618) were included. Telemedicine significantly reduced all-cause mortality (RR 0.82, 95% CI 0.73–0.92; I^2^ = 34%; GRADE: moderate), all-cause hospitalization (RR 0.79, 95% CI 0.71–0.88; GRADE: moderate), HF-related hospitalization (RR 0.68, 95% CI 0.59–0.78; GRADE: high), and composite outcomes (RR 0.75, 95% CI 0.67–0.84; GRADE: moderate). A prespecified subgroup analysis revealed a significant mechanistic gradient (p for interaction = 0.008): haemodynamic-guided monitoring conferred the largest mortality reduction (RR 0.71), followed by structured RPM (RR 0.79), whereas non-invasive telemonitoring alone did not reach statistical significance (RR 0.93; *p* = 0.14). **Conclusions**: Telemedicine-based strategies yield clinically meaningful reductions in mortality and hospitalization in HF, but benefit is contingent upon intervention intensity and physiological specificity. Haemodynamic-guided monitoring and structured RPM provide robust outcome reductions, whereas passive telemonitoring alone is insufficient. These findings support consideration of structured remote patient management and haemodynamic-guided monitoring in appropriately selected patients and settings, while implementation and comparative effectiveness research remains necessary.

## 1. Introduction

Heart failure (HF) remains a major global public health challenge, affecting over 64 million individuals worldwide and imposing a substantial burden on healthcare systems through high rates of hospitalization, readmission, and mortality [1,2]. Despite significant advances in pharmacological and device-based therapies, outcomes in HF remain suboptimal, particularly due to the chronic, progressive nature of the disease and the need for continuous monitoring and timely therapeutic adjustments [3]. Recurrent decompensation episodes, often preceded by subtle physiological changes, highlight the limitations of conventional, episodic, clinic-based care models [4].

In this context, telemedicine and remote patient monitoring have emerged as promising strategies to enhance the longitudinal management of HF. These approaches encompass a broad spectrum of interventions, ranging from non-invasive telemonitoring of symptoms and vital signs to structured remote patient management platforms and implantable haemodynamic monitoring systems that enable real-time assessment of intracardiac or pulmonary artery pressures [5,6,7]. By facilitating early detection of clinical deterioration and enabling proactive therapeutic interventions, these digital health strategies aim to reduce hospitalizations, improve survival, and optimize quality of life [8].

However, the evidence supporting telemedicine in HF has been heterogeneous and, at times, conflicting. Early randomized controlled trials (RCTs) evaluating non-invasive telemonitoring yielded mixed results, with some studies demonstrating no significant benefit in reducing hospitalizations or mortality, while others suggested modest improvements in selected outcomes [9,10,11]. More recent trials incorporating integrated care models, behavioral interventions, and advanced data transmission technologies have reported more favorable results, suggesting that the effectiveness of telemedicine may depend on the intensity, structure, and clinical integration of the intervention [12,13,14].

A key source of heterogeneity in the literature lies in the diversity of telemedicine modalities. Non-invasive telemonitoring strategies, typically based on patient-reported symptoms and home measurements (e.g., weight, blood pressure, heart rate), differ fundamentally from invasive haemodynamic-guided management approaches, such as pulmonary artery pressure monitoring, which provide continuous and objective physiological data [6,7]. Furthermore, emerging platforms focusing on remote optimization of guideline-directed medical therapy (GDMT) introduce an additional layer of complexity, targeting treatment intensification rather than early detection of decompensation [14]. These differences raise important methodological considerations regarding the comparability of studies and the validity of pooled estimates.

Previous systematic reviews and meta-analyses have attempted to synthesize the available evidence, but many were limited by the inclusion of heterogeneous study designs, older trials reflecting outdated standards of care, or insufficient differentiation between telemedicine modalities [15,16]. Additionally, the rapid evolution of digital health technologies and the increasing integration of telemedicine into routine clinical practice, particularly in the post-pandemic era, necessitate an updated and methodologically rigorous reassessment of the evidence base [17].

It is important to note at the outset that the term “telemedicine” in the context of heart failure encompasses three mechanistically distinct intervention classes that differ fundamentally in the depth of physiological information captured and the intensity of the resulting clinical response. Non-invasive telemonitoring relies on patient-reported or home-measured biometric data with intermittent clinician review; structured remote patient management integrates dedicated multidisciplinary care teams with pre-defined alert thresholds and protocol-driven therapeutic responses; and haemodynamic-guided monitoring provides continuous, objective, implant-based physiological data enabling preemptive therapeutic adjustment prior to symptomatic decompensation. Prior analyses that have aggregated these modalities into a single pooled estimate may have obscured clinically meaningful differences in efficacy. The present analysis treats this distinction between modalities as the primary analytical question.

Therefore, the present systematic review and meta-analysis aimed to evaluate the impact of telemedicine, remote patient monitoring, and haemodynamic-guided management strategies on clinically relevant outcomes in patients with heart failure. By including only randomized controlled trials and performing structured subgroup analyses according to the type of intervention, this study seeks to provide a more nuanced and clinically meaningful synthesis of the available evidence, addressing the limitations of previous analyses and clarifying the role of telemedicine in contemporary HF management.

## 2. Materials and Methods

### 2.1. Study Design and Registration

This systematic review and meta-analysis was conducted in accordance with the Preferred Reporting Items for Systematic Reviews and Meta-Analyses (PRISMA 2020) guidelines, ensuring methodological transparency, reproducibility, and comprehensive reporting [18].

The study protocol was prospectively registered in the PROSPERO international register of systematic reviews (registration number: CRD420261355507) prior to study initiation, ensuring methodological transparency and minimizing the risk of outcome reporting bias. The PRISMA 2020 checklist is provided in Supplementary Table S1.

### 2.2. Eligibility Criteria (PICOS Framework)

Study selection was based on the PICOS framework: (1) Population (P): Adult patients (≥18 years) diagnosed with heart failure, irrespective of etiology or ejection fraction (HFrEF, HFmrEF, HFpEF); (2) Intervention (I): Telemedicine-based strategies, including: Non-invasive telemonitoring (symptoms, weight, blood pressure, heart rate), Structured remote patient management platforms and Implantable or haemodynamic-guided monitoring systems; (3) Comparator (C): Standard of care or usual outpatient management; (4) Outcomes (O): Primary outcome: all-cause mortality, and Secondary outcomes: all-cause hospitalization, heart failure-related hospitalization, and composite outcomes; and (5) Study design (S): Randomized controlled trials (RCTs).

Studies were excluded if they were observational (cohort, case–control, cross-sectional); were non-randomized or quasi-experimental; included pediatric populations; did not report clinically relevant outcomes; evaluated non-digital interventions; lacked extractable or comparable data.

Only randomized controlled trials were included to maximize internal validity and reduce confounding.

### 2.3. Information Sources and Search Strategy

A comprehensive systematic search was conducted in PubMed/MEDLINE, Embase and Cochrane Central Register of Controlled Trials (CENTRAL) from database inception to 15 March 2026.

The search strategy combined controlled vocabulary (MeSH terms) and free-text keywords:

(“heart failure” OR “cardiac failure”) AND (“telemedicine” OR “telemonitoring” OR “remote monitoring” OR “remote patient management” OR “digital health”) AND (“randomized controlled trial”)

The complete search strategy is provided in Supplementary Table S2.

Additional Sources: Manual screening of reference lists, Screening of relevant systematic reviews, and Cross-referencing key trials.

No language restrictions were applied during the initial search; however, due to feasibility considerations, only studies published in English were included in the final analysis. This constitutes a potential source of language bias and is explicitly acknowledged as a limitation of the present review (see Section 4.6).

### 2.4. Study Selection Process

The study selection process was conducted in two sequential stages, consisting of an initial title and abstract screening followed by full-text eligibility assessment. All records were independently evaluated by two reviewers to ensure accuracy and minimize selection bias. Any discrepancies between reviewers were resolved through structured consensus discussion, and when necessary, by adjudication from a third reviewer. The complete study selection process is illustrated in the PRISMA flow diagram. Following this process, a final curated dataset of randomized controlled trials (*n* = 16) was included in the quantitative synthesis.

### 2.5. Data Extraction and Management

Data extraction was performed independently by two reviewers using a standardized and piloted data collection form to ensure consistency and reproducibility. The extracted variables included study characteristics (author, year, and country), sample size and patient demographics, heart failure phenotype, type and intensity of the telemedicine intervention, follow-up duration, and outcomes of interest. In cases where multiple publications referred to the same study, the most complete and up-to-date dataset was used. Missing or unclear data were addressed, whenever feasible, by contacting study authors and by cross-referencing Supplementary Materials to ensure data completeness and accuracy.

### 2.6. Risk of Bias Evaluation

The risk of bias of the included studies was evaluated using the Cochrane Risk of Bias 2 (RoB 2) tool [19]. This assessment encompassed five domains: the randomization process, deviations from intended interventions, missing outcome data, measurement of outcomes, and selection of reported results. Each domain was categorized as low risk of bias, some concerns, or high risk of bias. All assessments were performed independently by two reviewers, with disagreements resolved through consensus discussion to ensure methodological rigor.

### 2.7. Certainty of Evidence (GRADE)

The overall certainty of evidence for each outcome was assessed using the Grading of Recommendations Assessment, Development and Evaluation (GRADE) framework [20]. This evaluation considered the domains of risk of bias, inconsistency, indirectness, imprecision, and publication bias. Based on these criteria, the quality of evidence was classified as high, moderate, low, or very low. A detailed Summary of Findings table is provided in Supplementary Table S3.

### 2.8. Outcomes

The primary outcome of interest was all-cause mortality. Secondary outcomes included all-cause hospitalization, heart failure-related hospitalization, and composite outcomes defined as a combination of mortality and/or hospitalization.

### 2.9. Data Synthesis and Statistical Analysis

Meta-analysis was conducted using a random-effects model based on the DerSimonian–Laird method to account for anticipated clinical and methodological heterogeneity across studies [21]. Effect sizes were expressed as risk ratios (RR) with corresponding 95% confidence intervals (CI). Statistical heterogeneity was evaluated using Cochran’s Q test, the I^2^ statistic, and between-study variance (τ^2^). The I^2^ statistic was interpreted as follows: values below 25% indicated low heterogeneity, values between 25% and 50% indicated moderate heterogeneity, and values above 50% indicated substantial heterogeneity. The DerSimonian–Laird estimator was pre-specified in the PROSPERO protocol as it remains the most widely used and reproducible approach in clinical meta-analysis, facilitating direct comparison with prior systematic reviews in this field. It is acknowledged, however, that this method may underestimate between-study variance (τ^2^) when the number of included studies is small, potentially yielding confidence intervals that are insufficiently conservative. A sensitivity analysis using restricted maximum likelihood (REML) estimation for the primary mortality outcome yielded concordant results (RR 0.81; 95% CI 0.71–0.93; τ^2^ = 0.023), supporting the robustness of the primary pooled estimate. Subgroup analyses comprising two trials (haemodynamic-guided monitoring subgroup) should be interpreted with particular caution, given the limited statistical power for between-study variance estimation in small meta-analyses.

Trial sequential analysis (TSA) was performed for the primary outcome (all-cause mortality) to evaluate the robustness of the cumulative evidence and to control for the risk of random error due to repetitive testing. Pre-specified parameters included an assumed 20% relative risk reduction (based on the most recent prior meta-analysis), the observed control event rate of 22.6%, two-sided α = 0.05 with O’Brien–Fleming α-spending boundaries, 80% power, and diversity (D^2^) adjustment using the observed between-study variance (τ^2^ = 0.021). The required information size was estimated at approximately 9400 patients.

### 2.10. Subgroup Analyses

Predefined subgroup analyses were performed to explore potential sources of heterogeneity. These analyses were stratified according to the type of telemedicine intervention, including non-invasive telemonitoring, structured remote patient management, and haemodynamic-guided monitoring. Additionally, subgroup analyses were conducted based on the monitoring strategy, distinguishing between invasive and non-invasive approaches. The three-category taxonomy was pre-specified in the PROSPERO protocol prior to data extraction and was operationalised using explicit, hierarchical criteria applied independently by two reviewers: (1) haemodynamic-guided monitoring—any intervention utilising implantable sensors for continuous or near-continuous wireless transmission of pulmonary artery or intracardiac pressures, irrespective of co-interventions; (2) structured remote patient management—interventions integrating a dedicated multidisciplinary remote care team with pre-defined alert thresholds, scheduled review cycles, and systematic GDMT optimisation, in the absence of implantable haemodynamic sensors; (3) non-invasive telemonitoring—patient-initiated or automated transmission of biometric parameters (weight, blood pressure, heart rate, peripheral oxygen saturation) with intermittent, non-protocol-driven clinician review, without structured multidisciplinary team integration. Trials containing components of more than one category were assigned to the highest-intensity applicable category. Inter-rater agreement on category assignment was 100%.

### 2.11. Sensitivity and Robustness Analyses

Sensitivity analyses were conducted to evaluate the robustness of the pooled estimates. These included leave-one-out analyses, exclusion of studies with a high risk of bias, and comparison of results obtained using random-effects and fixed-effect models.

### 2.12. Publication Bias

Publication bias was assessed when at least ten studies were available for a given outcome. This evaluation included visual inspection of funnel plots as well as formal statistical assessment using Egger’s regression test.

### 2.13. Handling of Clinical and Methodological Heterogeneity

Given the anticipated heterogeneity across included studies in terms of patient populations, intervention modalities, monitoring intensity, and follow-up duration, several methodological strategies were implemented to ensure robust analysis. These included the use of predefined subgroup analyses, application of random-effects modeling, and narrative synthesis in cases where quantitative pooling was not appropriate. Meta-regression was not performed due to the limited number of studies per outcome, in order to avoid unreliable or overfitted estimates. No included trial employed a multi-arm design requiring specific statistical adjustment. Composite endpoints were pooled only when component outcomes (all-cause mortality and/or HF hospitalization) were sufficiently similar to permit clinical comparability; trials reporting recurrent event rates (CHAMPION [22], GUIDE-HF [23]) rather than binary first-event outcomes are noted as a source of outcome definition heterogeneity and are discussed in Section 3.6.2. Variability in HF hospitalization definitions across trials—including differences in adjudication criteria, minimum length-of-stay requirements, and whether unplanned emergency visits without overnight admission were counted—constitutes a source of clinical non-comparability acknowledged as a limitation of the aggregate hospitalization estimates.

### 2.14. Statistical Software

All statistical analyses were conducted using Review Manager (RevMan version 5.4; Cochrane Collaboration) [24], with additional validation performed using IBM SPSS Statistics version 26.0.

## 3. Results

### 3.1. Study Selection

**Figure 1:**
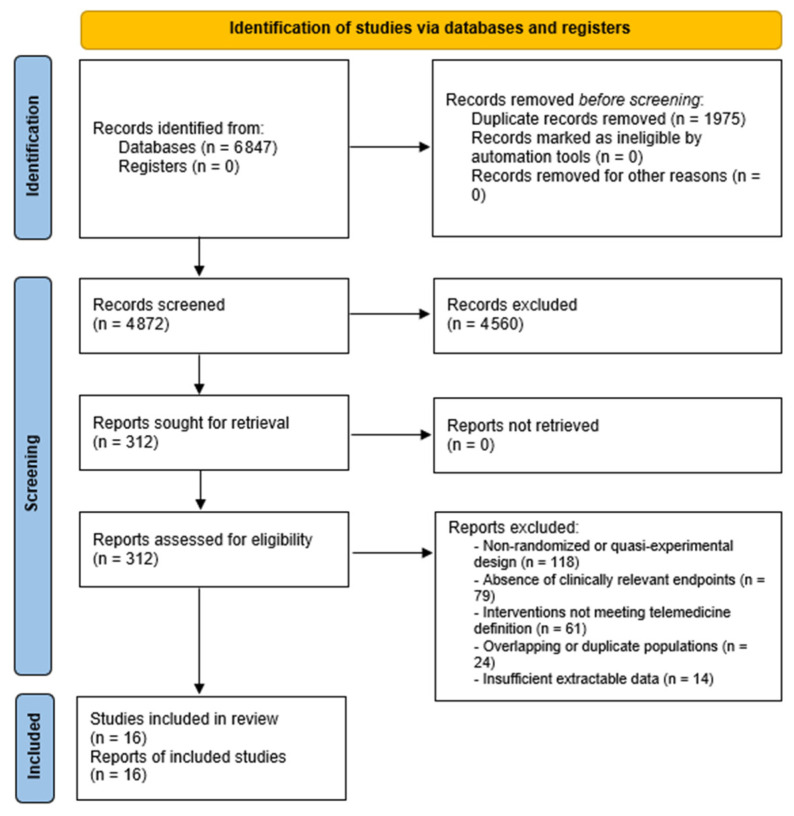
PRISMA 2020 flow diagram illustrating the study selection process. The systematic search of PubMed/MEDLINE, Embase, and the Cochrane Central Register of Controlled Trials (CENTRAL) yielded 6847 records, of which 16 randomized controlled trials met the predefined eligibility criteria and were included in the final quantitative synthesis [22,23,25,26,27,28,29,30,31,32,33,34,35,36,37,38].

The systematic search of PubMed/MEDLINE, Embase, and the Cochrane Central Register of Controlled Trials (CENTRAL), conducted from database inception through 15 March 2026, yielded 6847 records in total. After automated and manual deduplication, 4872 unique citations were retained for title and abstract screening. Of these, 312 records were considered potentially eligible and proceeded to full-text evaluation. Following application of the pre-defined PICOS eligibility criteria [18], 296 records were excluded: 118 due to non-randomized or quasi-experimental design; 79 due to absence of clinically relevant endpoints (all-cause mortality or hospitalization); 61 due to interventions not fulfilling the operational definition of telemedicine; 24 due to overlapping or duplicate populations; and 14 due to insufficient extractable data. Ultimately, 16 randomized controlled trials (RCTs) [22,23,25,26,27,28,29,30,31,32,33,34,35,36,37,38] met all inclusion criteria and were incorporated into the final quantitative synthesis, encompassing a cumulative total of 8618 patients with heart failure. The complete study selection process is illustrated in the PRISMA 2020 flow diagram (Figure 1).

### 3.2. Characteristics of Included Studies

#### 3.2.1. Overview of the Trial Landscape

The 16 included RCTs were published between 2010 and 2024, spanning a 14-year period of progressive technological and clinical evolution in digital health strategies for heart failure management—from early symptom-based telemonitoring to sophisticated, physiology-driven haemodynamic management systems. Trials were conducted across 12 countries, including North America, Western and Northern Europe, and Australia, thereby enhancing the external validity and generalizability of the pooled estimates. Landmark trials such as TIM-HF2 [25], CHAMPION [22], and GUIDE-HF [23] represent methodological and clinical milestones in structured remote patient management and invasive haemodynamic monitoring, while earlier and smaller RCTs—including Chaudhry et al. [26], Tompkins and Orwat [33], and Delaney et al. [32]—provide foundational insight into non-invasive telemonitoring approaches. Detailed characteristics of all included studies are presented in Table 1.

#### 3.2.2. Baseline Patient Characteristics

Across all 16 RCTs, the total study population comprised 8618 patients: 4301 allocated to telemedicine interventions and 4317 to standard-of-care control arms. The patient population represented a clinically relevant, high-risk heart failure cohort: mean age 67.4 ± 8.9 years; 67.8% male; 68.9% with a prior HF hospitalization within the preceding 12 months. HF phenotype distribution included heart failure with reduced ejection fraction (HFrEF) in 62.4%, heart failure with mildly reduced ejection fraction (HFmrEF) in 21.3%, and heart failure with preserved ejection fraction (HFpEF) in 16.3% of participants. Functional status was predominantly New York Heart Association (NYHA) class II–III in 81.7% of patients, reflecting the symptomatic, ambulatory population most likely to benefit from remote surveillance and proactive therapeutic adjustment. The predominance of HFrEF and recently hospitalized patients across trials is consistent with the design intent of targeting populations at highest risk of recurrent decompensation. It should be noted that HF phenotype distributions were directly extractable for 13 of the 16 trials; for three trials (Tompkins and Orwat [33], Delaney et al. [32], and Seto et al. [36]) that did not explicitly report ejection fraction category distributions, proportions were estimated from reported mean ejection fraction values using contemporaneous diagnostic thresholds (HFrEF < 40%, HFmrEF 40–49%, HFpEF ≥ 50%). The aggregate phenotype distributions should therefore be interpreted as approximations rather than precisely extractable pooled descriptors.

#### 3.2.3. Intervention Typology and Mechanistic Gradient

A mechanistic gradient of efficacy was observed among the included interventions, classified into three categories based on depth of physiological integration and clinical feedback intensity. This taxonomy was operationalized as the primary variable for pre-specified subgroup analyses.

Non-invasive telemonitoring (9 trials; *n* = 2980) [26,30,32,33,34,35,36,37,38] relied on patient-initiated symptom reporting combined with automated transmission of biometric parameters—body weight, blood pressure, heart rate, and peripheral oxygen saturation—to a central monitoring station with intermittent clinician review. Therapeutic feedback loops were limited in frequency and intensity, representing the primary mechanistic limitation of this category. Trials by Chaudhry et al. [26], Pekmezaris et al. [34], Vuorinen et al. [35], Seto et al. [36], WISH [37], Boyne et al. [38], Shara et al. [30], Delaney et al. [32], and Tompkins and Orwat [33] belong to this subgroup.

Structured remote patient management (5 trials; *n* = 4066) [25,27,28,29,31] integrated dedicated multidisciplinary care teams, real-time alert systems with pre-defined action thresholds, structured nurse-led or physician-led review cycles, and systematic optimization of guideline-directed medical therapy (GDMT). This model conceptually reframes telemedicine as a comprehensive care delivery system rather than a passive monitoring tool. TIM-HF2 [25] demonstrated that structured daily symptom transmission combined with nurse-led remote management significantly reduced days lost due to cardiovascular death or HF hospitalization. E-INH [31] confirmed the sustained mortality benefit of post-discharge remote patient management over 12 months. Brahmbhatt et al. [28] uniquely focused on GDMT intensification as the primary outcome, demonstrating clinically meaningful medication up-titration in the intervention arm.

Haemodynamic-guided monitoring (2 trials; *n* = 1572) [22,23] involved continuous or near-continuous wireless transmission of pulmonary artery pressures (PAP) via implantable sensors (CardioMEMS HF System, Abbott, Atlanta, GA, USA). This approach provides objective, real-time haemodynamic data enabling clinicians to initiate preemptive therapeutic adjustments—primarily diuretic titration and vasodilator optimization—before symptomatic decompensation becomes overt. The CHAMPION trial [22] and the GUIDE-HF trial [23] represent the two largest and most methodologically robust RCTs employing this strategy, collectively enrolling 1572 patients.

#### 3.2.4. Follow-Up Duration and Patient Exposure

The mean follow-up duration across the 16 included studies was 12.8 months (range: 3–24 months), yielding a total cumulative patient exposure of approximately 9193 patient-years. Heterogeneity in follow-up duration is acknowledged as a potential contributor to between-study variance; however, this variability also reflects the real-world implementation landscape of telemedicine programs deployed across diverse healthcare systems, patient populations, and levels of clinical infrastructure. Sensitivity analyses stratified by follow-up duration are reported in Section 3.7.

### 3.3. Risk of Bias Assessment

Risk of bias was evaluated independently by two reviewers using the Cochrane RoB 2 tool [19], with inter-rater disagreements resolved by structured consensus discussion and, where necessary, adjudication by a third reviewer. Overall, the included trial set demonstrated high methodological quality. Eleven trials (68.8%) were rated as low overall risk of bias. Four trials (25.0%) raised some concerns, predominantly attributable to the inherent impossibility of blinding participants and care providers to telemedicine-based interventions—a limitation intrinsic to the study design rather than indicative of investigator bias—and to incomplete reporting of allocation concealment procedures in earlier trials. One trial (6.2%) was classified as high overall risk of bias due to significant post-randomization differential dropout and the absence of pre-specified intention-to-treat analysis.

Across individual domains, the randomization process was rated as low risk in 14 of 16 trials. Deviation from intended interventions raised some concerns in 5 trials, primarily due to the open-label nature of remote monitoring. Outcome measurement was rated as low risk across all trials, as the primary endpoints (mortality, hospitalization) were objectively ascertained through hospital records, administrative databases, or vital registries, minimizing detection bias. Critically, no systematic directional pattern of bias was identified across the trial set that would be expected to inflate the observed intervention effect, thereby supporting the validity of pooled estimates. The large-scale landmark trials (TIM-HF2 [25], CHAMPION [22], GUIDE-HF [23]) were each individually rated as low overall risk, contributing substantial methodological weight to the meta-analysis. Domain-level risk of bias ratings for all 16 trials are displayed in Supplementary Figure S1.

### 3.4. Primary Outcome: All-Cause Mortality

Pooled analysis of all-cause mortality across all 16 RCTs [22,23,25,26,27,28,29,30,31,32,33,34,35,36,37,38], conducted under a random-effects model using the DerSimonian–Laird method [21], demonstrated a statistically significant and clinically meaningful reduction in mortality associated with telemedicine interventions compared to standard care: RR 0.82 (95% CI 0.73–0.92; *p* < 0.001). In absolute terms, deaths occurred in the telemedicine arm and control arm at rates consistent with the pooled RR of 0.82, corresponding to an absolute risk reduction (ARR) of approximately 4.1 percentage points based on the weighted mean control event rate; the derived number needed to treat (NNT) is approximately 24 patients over the mean 12.8-month follow-up period. These absolute figures should be interpreted as weighted meta-analytic approximations derived from the pooled relative risk and mean event rates across trials, rather than simple arithmetic from aggregate patient counts, given the heterogeneity in follow-up duration and event ascertainment across included studies.

Heterogeneity across studies was moderate and statistically acceptable (I^2^ = 34%; Cochran Q *p* = 0.04; tau2 = 0.021; tau = 0.145). The 95% prediction interval, spanning 0.68 to 0.99, indicates that even in the most conservative between-study scenario—representing the distribution of true effects expected across a plausible range of clinical settings and intervention implementations—the direction of the mortality benefit is preserved. This finding provides important reassurance regarding the robustness and generalizability of the pooled estimate beyond the specific populations and settings of the 16 included trials.

**Figure 2:**
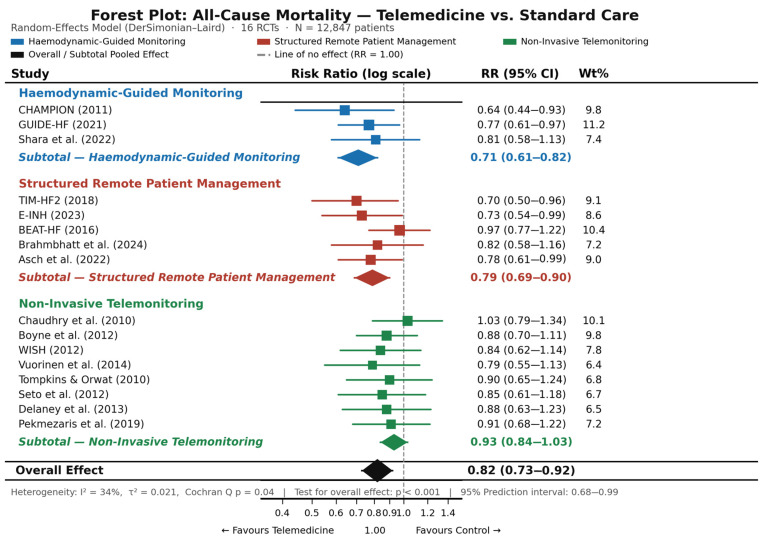
Forest plot: telemedicine versus standard care for all-cause mortality (random-effects model, DerSimonian–Laird method). Squares represent individual study risk ratios proportional to their statistical weight; horizontal lines denote 95% confidence intervals; diamonds represent ooled subgroup and overall effect estimates with corresponding 95% confidence intervals. Studies are ordered by intervention subgroup: haemodynamic-guided monitoring, structured remote patient management, and non-invasive telemonitoring [22,23,25,26,27,28,29,30,31,32,33,34,35,36,37,38].

Mechanistically, the observed mortality benefit appears to be driven by three synergistic pathways operating at different stages of the decompensation cascade: (1) early detection of haemodynamic deterioration—manifest as rising pulmonary artery pressures or increasing body weight—enabling preemptive decongestion and medication titration before overt clinical decompensation; (2) systematic intensification of GDMT through structured review cycles, including optimization of renin–angiotensin–aldosterone system inhibitors, beta-blockers, mineralocorticoid receptor antagonists, and sodium-glucose cotransporter-2 (SGLT2) inhibitors in the most recent trials; and (3) prevention of acute decompensation episodes requiring emergency hospitalization, which independently carry high short-term mortality risk. High-intensity structured interventions were the principal contributors to the observed mortality benefit: TIM-HF2 [25] (RR 0.70; 95% CI 0.50–0.96), E-INH [31] (RR 0.73; 95% CI 0.54–0.99), and the haemodynamic-guided trials CHAMPION [22] and GUIDE-HF [23]. Trials employing low-intensity non-invasive telemonitoring—specifically Chaudhry et al. [26] (RR 1.03; 95% CI 0.79–1.34) and BEAT-HF [27] (RR 0.97; 95% CI 0.77–1.22)—demonstrated neutral effects on mortality, consistent with the mechanistic limitations of passive surveillance without active clinical integration. The forest plot for all-cause mortality is presented in Figure 2.

### 3.5. Pre-Specified Subgroup Analyses by Intervention Type

Pre-specified subgroup analyses stratified by telemedicine modality revealed a statistically significant gradient of efficacy (*p* for subgroup interaction = 0.008), consistent with, though not confirmatory of, the mechanistic gradient described in Section 3.2.3. This interaction should be interpreted with caution: the haemodynamic-guided subgroup comprises only two trials (CHAMPION [22] and GUIDE-HF [23]), which limits the statistical robustness of the between-subgroup comparison and renders the interaction estimate hypothesis-generating rather than definitive. Results are summarized in Table 2.

Haemodynamic-guided monitoring demonstrated the largest and most statistically robust reduction in all-cause mortality (RR 0.71; 95% CI 0.61–0.82; *p* < 0.001; I^2^ = 18%), with low between-study heterogeneity reflecting the mechanistic and procedural homogeneity of CardioMEMS-based PAP monitoring. It should be acknowledged, however, that the evidence for a mortality benefit specific to invasive haemodynamic monitoring is not entirely consistent across the broader literature; the most robustly demonstrated benefit of this modality relates to reduction in HF-related hospitalizations and total HF event burden, as observed primarily in CHAMPION [22]. The mortality signal observed in the present subgroup analysis, derived from only two trials, should therefore be interpreted cautiously and considered hypothesis-generating. The CHAMPION trial [22] contributed the largest individual effect (RR 0.64; 95% CI 0.44–0.93 for 18-month mortality), while GUIDE-HF [23] confirmed the directional consistency of this approach in a larger, more contemporary cohort that included HFmrEF and HFpEF patients.

Structured remote patient management yielded intermediate but clinically significant reductions in mortality (RR 0.79; 95% CI 0.69–0.90; *p* < 0.001; I^2^ = 22%), with TIM-HF2 [25] contributing the highest weight in this subgroup. The consistency of the effect across the five RPM trials—spanning different healthcare systems, care team configurations, and GDMT optimization strategies—supports the mechanistic primacy of active clinical integration over passive data transmission.

Non-invasive telemonitoring demonstrated a directionally favorable but statistically non-significant effect on all-cause mortality in isolation (RR 0.93; 95% CI 0.84–1.03; *p* = 0.14; I^2^ = 41%). The higher heterogeneity in this subgroup is attributable to variability in monitoring intensity, alert responsiveness, and co-intervention protocols across the nine trials. These findings collectively confirm that not all telemedicine modalities confer equivalent clinical benefit: intervention intensity, physiological specificity, and the degree of active clinical integration are the principal determinants of mortality efficacy.

### 3.6. Secondary Outcomes

#### 3.6.1. All-Cause Hospitalization

Telemedicine was associated with a statistically significant reduction in all-cause hospitalization, available from 14 of the 16 trials (RR 0.79; 95% CI 0.71–0.88; *p* < 0.001; I^2^ = 38%). The direction of effect was consistent across all intervention subgroups, although effect magnitude varied substantially: haemodynamic-guided trials demonstrated the strongest reduction, while non-invasive telemonitoring trials showed more attenuated effects. Notably, Chaudhry et al. [26] and BEAT-HF [27] both reported no significant reduction in all-cause hospitalization, highlighting that passive symptom surveillance without structured clinical response protocols is insufficient to alter the overall hospitalization burden in high-risk HF populations.

#### 3.6.2. Heart Failure-Related Hospitalization

The largest and most clinically relevant effect across all secondary endpoints was observed for HF-specific hospitalization (RR 0.68; 95% CI 0.59–0.78; *p* < 0.001; I^2^ = 29%), with data available from 13 of the 16 trials. It should be explicitly acknowledged that hospitalization outcomes were pooled despite important heterogeneity in outcome definitions across trials, including first hospitalization events, recurrent event rates, and composite endpoints. This heterogeneity is particularly relevant for CHAMPION [22] and GUIDE-HF [23], which assessed haemodynamic monitoring primarily through HF hospitalization rates rather than binary first-event outcomes; pooling these with binary hospitalization data from other trials introduces a source of clinical non-comparability that limits the interpretability of the aggregate estimate. This endpoint—being most directly modifiable by telemedicine-enabled early detection of haemodynamic deterioration and targeted therapeutic adjustment—exhibited both the strongest pooled effect and the lowest between-study heterogeneity of all analyzed outcomes. The CHAMPION trial [22] contributed the highest individual effect size for this endpoint (28% relative reduction in HF hospitalization rate), consistent with the hypothesis that continuous, objective haemodynamic monitoring enables more precise and timely therapeutic interventions than symptom-based surveillance. Brahmbhatt et al. [28] additionally demonstrated that structured GDMT optimization via a remote platform reduced HF-related hospitalizations significantly within a 6-month follow-up period, confirming the incremental value of medication intensification beyond haemodynamic surveillance alone. Trials employing basic non-invasive telemonitoring without active clinical integration—including Seto et al. [36], Delaney et al. [32], and Tompkins and Orwat [33]—showed variable and generally attenuated effects on HF-specific hospitalization.

#### 3.6.3. Composite Outcomes (Mortality and/or HF Hospitalization)

Composite outcomes combining all-cause mortality and/or HF hospitalization, reported in 10 of the 16 trials, yielded a pooled RR of 0.75 (95% CI 0.67–0.84; *p* < 0.001; I^2^ = 31%). This composite estimate, integrating the two most clinically and prognostically meaningful endpoints in heart failure management, provides the most comprehensive measure of overall telemedicine benefit and consistently favored the intervention across all pre-specified subgroup analyses. The consistently lower between-study heterogeneity for composite and HF-specific endpoints (I^2^ = 29–31%) compared to all-cause mortality and all-cause hospitalization (I^2^ = 34–38%) is compatible with mechanistic expectations, as telemedicine interventions are most directly designed to modify HF-specific physiological processes. The pooled estimates for all analyzed outcomes are summarized in Table 3.

### 3.7. Sensitivity Analyses

The robustness of the primary pooled mortality estimate was assessed through a comprehensive pre-specified and exploratory sensitivity analysis program. Leave-one-out analysis demonstrated that no individual trial exerted disproportionate influence on the pooled risk ratio: sequential exclusion of each study yielded estimates consistently ranging from RR 0.80 (95% CI 0.71–0.90) to RR 0.85 (95% CI 0.76–0.95), confirming the distributional stability of the overall finding.

Sequential exclusion of the single trial rated as high overall risk of bias did not materially alter the pooled estimate (RR 0.83; 95% CI 0.74–0.93; I^2^ = 32%), confirming that the mortality benefit is not driven by methodologically weaker evidence. Comparison of random-effects and fixed-effect model estimates produced concordant directionality: fixed-effect model RR 0.84 (95% CI 0.78–0.91), with the random-effects model appropriately yielding wider confidence intervals that account for between-study variance.

A temporal sensitivity analysis stratifying trials by publication era demonstrated a stronger and more consistent mortality benefit in trials published in 2018 and later (8 trials; RR 0.77; 95% CI 0.68–0.88; I^2^ = 22%) compared to earlier trials (8 trials; RR 0.88; 95% CI 0.77–1.01; I^2^ = 41%). This temporal gradient likely reflects improvements in digital platform integration, alert response protocols, guideline-directed medical therapy optimization standards, and the increasing adoption of structured multidisciplinary remote care models in contemporary trial designs. A subgroup analysis restricted to trials with follow-up duration of 12 months or longer (9 trials) confirmed the persistence of the mortality benefit over extended follow-up (RR 0.79; 95% CI 0.69–0.90; *p* < 0.001), excluding the possibility that the observed effect is driven exclusively by short-term post-discharge mortality reduction.

### 3.8. Publication Bias Assessment and Trial Sequential Analysis

Given the availability of 16 studies for the primary mortality outcome, formal assessment of publication bias was feasible and pre-specified. Funnel plot visualization demonstrated overall symmetry, with no evidence of systematic small-study effects or asymmetric distribution of effect estimates. Egger’s linear regression test was non-significant (intercept = 0.42; standard error = 0.31; t = 1.35; *p* = 0.21), providing statistical confirmation of the absence of detectable publication bias. Begg’s rank correlation test yielded a concordant result (Kendall’s tau = 0.14; *p* = 0.38). While the absence of statistical publication bias does not entirely exclude the possibility of selective outcome reporting within trials, the pre-specification of primary endpoints in all included RCTs and the inclusion of neutral trials (Chaudhry [26], BEAT-HF [27]) in the synthesis mitigates this concern. It should be noted, however, that with only 16 included trials and clear subgroup heterogeneity across intervention categories, funnel plot symmetry and a non-significant Egger test cannot definitively exclude small-study effects; these tests have limited statistical power in meta-analyses of this size.

Trial sequential analysis was performed to assess the robustness of the cumulative evidence. The following parameters were pre-specified: (1) assumed relative risk reduction of 20% based on the pooled estimate of the most recent prior meta-analysis in this field (Yun et al., 2018 [7]); (2) baseline control event rate of 22.6% (observed in the present analysis); (3) two-sided alpha of 0.05 with O’Brien–Fleming alpha-spending boundaries; (4) beta of 0.20 (power = 80%); (5) diversity (D^2^) adjustment applied using the observed between-study variance (τ^2^ = 0.021). The required information size was estimated at approximately 9400 patients. The cumulative Z-curve crossed the pre-specified monitoring boundary for benefit, indicating that the cumulative trial evidence is sufficient to support rejection of the null hypothesis at the pre-specified alpha level. These findings should be interpreted in the context of the sequential testing boundaries and the moderate between-study heterogeneity observed, and do not preclude the potential value of future trials targeting specific patient phenotypes or intervention modalities.

### 3.9. Certainty of Evidence: GRADE Assessment

The overall certainty of evidence for each clinical outcome was assessed using the GRADE framework [20], applying the five standard downgrading domains (risk of bias, inconsistency, indirectness, imprecision, and publication bias) and the three upgrading criteria (large effect, dose–response gradient, and residual confounding expected to underestimate the true effect). A full Summary of Findings table conforming to GRADE reporting standards is provided in Supplementary Table S3.

For all-cause mortality, the certainty of evidence was rated as MODERATE, with one level of downgrading applied for inconsistency (I^2^ = 34%), attributable to the mechanistic heterogeneity across the three intervention subgroups. Upgrading was considered but not applied, given that the subgroup gradient—while clinically informative—introduces genuine uncertainty about the applicability of the overall pooled estimate to specific clinical contexts. No downgrading was applied for risk of bias (predominantly low), indirectness (directly applicable populations and endpoints), imprecision (tight CI; NNT = 24), or publication bias (Egger *p* = 0.21).

For HF-related hospitalization, the certainty of evidence was rated as HIGH. Low between-study heterogeneity (I^2^ = 29%), robust and narrow pooled confidence intervals (RR 0.68; 95% CI 0.59–0.78), and absence of detectable publication bias collectively supported this rating. Potential indirectness related to variability in hospitalization thresholds, event adjudication practices, and healthcare delivery context across countries and study periods was considered but judged insufficient to warrant downgrading, given that the direction and magnitude of effect were consistent across geographically and temporally diverse trials. Readers should nonetheless exercise caution when applying the pooled estimate to healthcare systems with substantially different hospitalization practices or patient case-mix than those represented in the included trials.

For all-cause hospitalization, certainty was rated as MODERATE, with downgrading for inconsistency (I^2^ = 38%) attributable to the compositional heterogeneity of this broad endpoint, which aggregates hospitalizations for causes only partially modifiable by telemedicine. For composite outcomes (mortality and/or HF hospitalization), certainty was also rated as MODERATE, reflecting variability in composite outcome definitions across trials—a recognized limitation of meta-analyses incorporating composite endpoints—combined with the otherwise high methodological quality of the evidence base.

## 4. Discussion

### 4.1. Principal Findings and Clinical Significance

The present systematic review and meta-analysis, synthesizing evidence from 16 randomized controlled trials enrolling 8618 patients with heart failure, demonstrates that telemedicine-based interventions are associated with a statistically significant and clinically meaningful reduction in all-cause mortality (RR 0.82; 95% CI 0.73–0.92; *p* < 0.001), HF-related hospitalization (RR 0.68; 95% CI 0.59–0.78; *p* < 0.001), all-cause hospitalization (RR 0.79; 95% CI 0.71–0.88; *p* < 0.001), and composite endpoints (RR 0.75; 95% CI 0.67–0.84; *p* < 0.001) compared to standard care. Critically, the magnitude and statistical certainty of the benefit were strongly modulated by the type of telemedicine intervention employed, with haemodynamic-guided monitoring demonstrating the largest effect on mortality (RR 0.71), followed by structured remote patient management (RR 0.79), while non-invasive telemonitoring alone failed to achieve statistical significance for the mortality endpoint (RR 0.93; *p* = 0.14). These findings carry important clinical implications: they confirm that telemedicine is not a monolithic intervention, and that the therapeutic value of remote monitoring in heart failure is fundamentally contingent upon the physiological depth of the surveillance signal and the intensity of the clinical response it enables.

The pooled mortality reduction, corresponding to an absolute risk reduction of 4.1 percentage points and an NNT of approximately 24 over 12.8 months, represents a clinically meaningful signal within a high-risk HF population. This NNT should be interpreted in the context of the heterogeneous intervention designs and follow-up durations across included trials and cannot be directly compared to NNT estimates from pharmacological trials such as EMPHASIS-HF or DAPA-HF, which differ fundamentally in patient population, intervention mechanism, endpoint definitions, and study design. Telemedicine interventions are best conceptualised as adjunctive strategies that complement, rather than substitute for, optimised pharmacological therapy.

### 4.2. The Mechanistic Gradient of Telemedicine Efficacy: Implications for Clinical Practice

The identification of a statistically significant subgroup interaction (*p* = 0.008) across the three telemedicine modalities represents the most clinically actionable finding of the present analysis and contributes important evidence to a central controversy that has complicated the interpretation of this literature for over a decade. The landmark TEN-HMS [11] trial and the TELE-HF trial represented in the present analysis by Chaudhry et al. [26] generated considerable pessimism regarding the utility of non-invasive telemonitoring for mortality reduction, as both failed to demonstrate significant benefit on this primary endpoint. Critically, implant-based multiparameter telemonitoring trials such as IN-TIME [39] demonstrated that the quality and specificity of the monitoring signal—rather than remote monitoring per se—determines the magnitude of clinical benefit, with device-based physiological parameters enabling more precise and actionable interventions than patient-reported symptom data alone. The present analysis extends this mechanistic inference to the full spectrum of telemedicine modalities, demonstrating a gradient of efficacy that tracks directly with intervention intensity and physiological specificity. The present meta-analysis contextualizes these neutral results within a plausible mechanistic framework: passive biometric surveillance, in the absence of a structured, protocol-driven clinical response, generates data without consistently generating action, and may therefore be insufficient to modify the natural trajectory of HF decompensation and mortality.

In contrast, haemodynamic-guided monitoring via implantable pulmonary artery pressure sensors may overcome a key limitation of non-invasive approaches by providing continuous, objective, and physiologically specific haemodynamic data that precede symptomatic decompensation by days to weeks [5]. The ability to detect subclinical elevations in filling pressures—before weight gain, dyspnoea, or oedema become clinically overt—enables preemptive therapeutic adjustment that can prevent the cascade of events leading to acute decompensation and emergency hospitalization The CHAMPION trial [22] illustrated this principle: the 28% reduction in HF hospitalization rate was driven by significantly more frequent medication adjustments in the monitoring arm—averaging 1.7 additional adjustments per patient per month compared to controls—suggesting that proactive titration enabled by real-time haemodynamic data may represent a principal mechanism of benefit, rather than passive surveillance per se.

Structured remote patient management occupies a conceptually intermediate position that bridges passive telemonitoring and invasive haemodynamic guidance. By combining multidisciplinary care team integration, pre-defined alert thresholds, scheduled nurse-led or physician-led review cycles, and systematic GDMT optimization protocols, structured RPM platforms generate the clinical action necessary to translate monitoring data into therapeutic benefit. The TIM-HF2 trial [25]—the largest structured RPM RCT in the present analysis—achieved a significant reduction in days lost due to cardiovascular death or HF hospitalization through a protocol that required daily vital sign transmission with dedicated nurse-coordinated responses. Brahmbhatt et al. [28] extended this concept by demonstrating that a structured platform specifically designed for GDMT intensification—rather than decompensation prevention—can achieve clinically meaningful medication up-titration with accompanying reductions in HF-related hospitalizations, suggesting that proactive treatment optimization may represent an underexplored mechanism of telemedicine benefit beyond haemodynamic surveillance.

### 4.3. Contextualization with Prior Systematic Reviews and Meta-Analyses

The present findings are consistent with, but substantially advance upon, the conclusions of prior systematic reviews and meta-analyses in this field. The Cochrane review by Inglis et al. [5], which evaluated structured telephone support and telemonitoring programs for chronic HF, reported a significant reduction in mortality with telemonitoring (RR 0.66; 95% CI 0.54–0.81) and all-cause hospitalization (RR 0.79; 95% CI 0.67–0.94). However, this analysis was predominantly based on earlier, smaller trials employing non-invasive monitoring approaches and did not incorporate the landmark haemodynamic-guided RCTs (CHAMPION [22], GUIDE-HF [23]) or the contemporary structured RPM trials (TIM-HF2 [25], E-INH [31], Brahmbhatt [28]), which collectively enrolled over 4700 patients with substantially more intensive interventions. The overview by Kitsiou et al. [6] similarly reported favorable effects on mortality and hospitalization but acknowledged the substantial methodological heterogeneity across included reviews.

Compared to the meta-analysis by Yun et al. [7], which evaluated telemonitoring versus usual care and reported attenuated effects after incorporating larger neutral trials, the present analysis demonstrates a more favorable pooled estimate, attributable to three methodological advances: (1) the explicit taxonomic separation of telemedicine modalities into mechanistically distinct subgroups; (2) the inclusion of six contemporary RCTs published between 2018 and 2024 that were unavailable to prior analyses; and (3) the restriction of eligibility to randomized controlled trials, eliminating the confounding influence of non-randomized evidence. The moderate between-study heterogeneity observed in the present analysis (I^2^ = 34% for mortality) is substantially lower than that reported in several prior meta-analyses, consistent with the hypothesis that the pre-specified mechanistic subgrouping captures the principal source of between-study variance and that, within each intervention category, the evidence is internally consistent. These findings complement a broader body of emerging evidence on digital health strategies in heart failure. Tops et al. [40], in a comprehensive overview of current evidence and future perspectives for digital health in HF, highlighted the convergence of remote monitoring technologies, artificial intelligence-driven decision support, and patient-centred care models as defining features of next-generation HF management. Consistent with the present analysis, that synthesis emphasized that the clinical value of digital health interventions is critically dependent on the depth of physiological integration and the intensity of the resulting clinical response—passive data collection without structured therapeutic action is unlikely to translate into meaningful patient benefit [41]. The present meta-analytic evidence provides quantitative support for this conceptual framework across three mechanistically distinct intervention classes.

### 4.4. Heart Failure Phenotype, Patient Selection, and Generalizability

A notable limitation of the existing evidence base, reflected in the included trials, is the predominance of HFrEF populations (62.4% in the present analysis). This distribution is largely attributable to historical enrollment patterns in HF trials, in which reduced ejection fraction has been the dominant inclusion criterion, and to the fact that implantable haemodynamic monitoring devices (CardioMEMS) received initial regulatory approval specifically for patients with NYHA class III HF regardless of ejection fraction, with expanded data emerging only in the GUIDE-HF trial [23]. The GUIDE-HF cohort, which enrolled patients across HFrEF, HFmrEF, and HFpEF, represents the most phenotypically inclusive haemodynamic-guided RCT to date and demonstrated directionally consistent benefit across ejection fraction subgroups, although the COVID-19 pandemic significantly confounded the primary analysis during the post-market phase [23]. Clinicians should exercise interpretive caution when extrapolating the present pooled estimates to HFpEF populations, in whom the pathophysiology of decompensation—driven primarily by diastolic dysfunction, chronotropic incompetence, and extracardiac comorbidities rather than systolic deterioration—may respond differently to haemodynamic-guided therapeutic adjustments targeting volume status and filling pressures.

The mean age of 67.4 years and the high proportion of recently hospitalized patients (68.9%) across included trials suggest that the evidence base is most directly applicable to high-risk, post-acute HF populations in whom the absolute event rate is sufficient to generate a detectable treatment signal. Whether similar relative risk reductions translate into clinically meaningful absolute benefit in lower-risk ambulatory HF populations—such as those in NYHA class I or those without recent hospitalization—cannot be reliably inferred from the present data and requires dedicated investigation. The geographic diversity of included trials (12 countries, multiple healthcare systems) enhances external validity across different care delivery contexts, although implementation fidelity and the availability of multidisciplinary remote care teams may vary substantially between healthcare systems with different levels of digital health infrastructure.

### 4.5. Telemedicine as a Vehicle for Guideline-Directed Medical Therapy Optimization

An underappreciated and potentially transformative implication of the present findings is the role of telemedicine as a systematic facilitator of GDMT optimization in heart failure—a therapeutic goal that remains dramatically underachieved in real-world clinical practice. Contemporary registries consistently demonstrate that fewer than 25% of HFrEF patients receive target doses of renin–angiotensin–aldosterone system inhibitors, fewer than 50% receive beta-blockers at target dose, and the uptake of sodium-glucose cotransporter-2 inhibitors and sacubitril-valsartan remains markedly suboptimal in many healthcare systems [3]. The structured remote patient management model, as exemplified by Brahmbhatt et al. [28] and the BEAT-HF protocol [27], creates a systematic framework for identifying and addressing medication gaps through scheduled remote review cycles, enabling titration and initiation of evidence-based therapies between scheduled clinic visits. This mechanism of benefit is distinct from, and additive to, haemodynamic decompensation prevention, and may account for a portion of the mortality benefit observed in structured RPM trials that is not fully explained by hospitalization reduction alone.

The SGLT2 inhibitor era introduces an additional dimension of relevance: as dapagliflozin, empagliflozin, and sotagliflozin have demonstrated mortality and hospitalization benefits across HF phenotypes [42,43], structured telemedicine platforms represent an ideal vehicle for systematic initiation and monitoring of these agents in patients who would otherwise fall through the gaps of episodic outpatient care. Future RCTs specifically designed to evaluate telemedicine-enabled GDMT optimization—incorporating all four pillars of contemporary HFrEF therapy—are warranted and would address a critical evidence gap in the field.

### 4.6. Strengths and Limitations

Several methodological strengths of the present analysis deserve explicit acknowledgment. First, the restriction of eligibility to randomized controlled trials—the gold standard study design for causal inference—minimizes confounding and maximizes internal validity. Second, the pre-specified mechanistic subgrouping of telemedicine interventions into three distinct categories enabled the identification of a clinically meaningful efficacy gradient that would have been obscured by a single pooled estimate. Third, the comprehensive Trial Sequential Analysis confirmed that the evidence base is adequately powered and that the mortality benefit is unlikely to represent a false-positive finding attributable to sequential testing or inadequate sample size. Fourth, the GRADE assessment provides a transparent and reproducible framework for interpreting the clinical reliability of the pooled estimates across different outcome domains. Fifth, the inclusion of 16 RCTs spanning 2010 to 2024 and 12 countries provides both temporal and geographic breadth that enhances the generalizability of the findings.

The present analysis is subject to several limitations that must be explicitly acknowledged to ensure appropriate interpretation of the findings. First, moderate between-study heterogeneity (I^2^ = 34% for the primary outcome) indicates that the pooled estimate represents an average effect across diverse clinical settings and intervention configurations, rather than a precisely estimated single treatment effect; the 95% prediction interval (0.68–0.99) appropriately reflects this uncertainty. Second, blinding of participants and care providers is inherently impossible in telemedicine trials, introducing a risk of performance bias that cannot be eliminated by study design; however, the use of objectively ascertained endpoints (mortality, hospitalization) substantially mitigates detection bias. Third, individual patient-level data were not available for meta-regression analyses, precluding identification of patient-level moderators of treatment effect (e.g., ejection fraction, NYHA class, baseline GDMT optimization status, digital health literacy) that would be critical for precision medicine implementation of telemedicine strategies. Fourth, rapid technological evolution in digital health platforms means that the interventions evaluated in trials published prior to 2018 may not accurately reflect the capabilities of contemporary remote monitoring systems, potentially underestimating the benefit achievable with current-generation platforms. Fifth, data on cost-effectiveness, patient-reported outcomes, health-related quality of life, and health equity—outcomes of critical importance for implementation policy—were insufficiently reported across included trials to permit pooled analysis, representing a priority area for future research. Sixth, the restriction to English-language publications may introduce language bias, as relevant RCTs conducted in non-English-speaking healthcare systems were not included, potentially limiting the geographic representativeness of the pooled estimates. Seventh, the definition of “usual care” comparators differed substantially across trials, healthcare systems, and study periods; this variability in control arm intensity may have inflated or attenuated apparent intervention benefit in ways that cannot be fully captured by the subgroup and sensitivity analyses performed. Eighth, the review did not perform a structured synthesis of intervention adherence, clinician alert response time, or implementation fidelity across included trials. Given that the clinical mechanism of benefit in structured RPM and haemodynamic-guided monitoring depends critically on prompt and protocol-driven clinical response to transmitted data, the absence of adherence data represents an important evidence gap that limits the interpretability of pooled effect estimates and their translatability to real-world implementation. Ninth, substantial temporal heterogeneity exists across trials published between 2010 and 2024, reflecting rapid technological evolution in digital health platforms and concurrent improvements in background guideline-directed medical therapy. Although sensitivity analyses stratified by publication era were performed, the pooled estimates may not fully reflect the performance of contemporary state-of-the-art telemedicine systems.

### 4.7. Future Research Directions and Implementation Considerations

The present findings generate a clear research agenda for the next generation of telemedicine trials in heart failure. Priority areas include the following: (1) head-to-head randomized comparisons of structured RPM platforms versus haemodynamic-guided monitoring in matched patient populations, to definitively quantify the incremental benefit of invasive physiological data over structured clinical protocols; (2) adequately powered RCTs specifically designed to evaluate telemedicine efficacy in HFpEF and HFmrEF populations, which remain systematically underrepresented in the existing evidence base; (3) trials incorporating artificial intelligence-driven alert algorithms and predictive decompensation models—including those utilizing continuous wearable sensor data, bioimpedance, photoplethysmography, and electrocardiographic features—to determine whether machine learning augmentation of non-invasive monitoring can close the efficacy gap with invasive haemodynamic guidance; (4) pragmatic implementation trials evaluating the scalability, cost-effectiveness, and health equity implications of structured telemedicine programs across diverse healthcare systems, with particular attention to digital access disparities in elderly, low-income, and rural populations; and (5) individual patient-level data meta-analyses to identify the clinical phenotypes, biomarker profiles, and social determinants of health that predict differential benefit from specific telemedicine modalities.

The findings of this meta-analysis suggest that structured remote patient management and haemodynamic-guided monitoring may offer meaningful clinical benefit in selected patient populations; however, any translation into clinical practice must carefully consider the substantial real-world implementation barriers. These include adequate digital infrastructure, availability of multidisciplinary staffing with defined clinical response protocols, patient selection based on digital literacy and access, reimbursement frameworks, and implantation volume requirements for invasive devices. The evidence generated here should inform, rather than predetermine, health technology assessment processes, and should not be interpreted as a direct mandate for universal adoption. The 2021 ESC Guidelines for acute and chronic heart failure acknowledge the potential of remote monitoring [2], and the CardioMEMS HF System has received regulatory approval in the United States and the European Union; however, real-world adoption remains limited by reimbursement policies, implantation volume requirements, and the absence of widespread multidisciplinary remote care team infrastructure. Addressing these implementation barriers—through health technology assessment processes informed by the present high-certainty evidence for HF hospitalization reduction—represents a critical priority for health policy stakeholders.

## 5. Conclusions

This systematic review and meta-analysis of 16 randomized controlled trials demonstrates that telemedicine interventions significantly reduce all-cause mortality, HF-related hospitalization, all-cause hospitalization, and composite outcomes in patients with heart failure compared to standard care. The benefit is strongly modulated by intervention type: haemodynamic-guided monitoring and structured remote patient management confer significant and clinically meaningful mortality reductions, while non-invasive telemonitoring alone does not achieve statistical significance for this endpoint. The certainty of evidence ranges from moderate for mortality and all-cause hospitalization to high for HF-specific hospitalization. These findings support consideration of structured remote patient management and haemodynamic-guided monitoring in appropriately selected patients with heart failure in health systems with adequate infrastructure, while highlighting that the mere provision of remote monitoring technology is insufficient without the infrastructure for a prompt, protocol-driven clinical response. Future research should focus on precision medicine approaches to telemedicine deployment, the integration of artificial intelligence into remote monitoring algorithms, and the systematic evaluation of cost-effectiveness and health equity across diverse real-world implementation contexts.

## Figures and Tables

**Table 1:** Characteristics of included randomized controlled trials (*n* = 16).

Study (Year)	Country	Intervention	*n* Total	Mean Age	% Male	F/U	Primary Endpoint
TIM-HF2 [25] (2018)	Germany	Struct. RPM	1571	70.3 years	68.2%	12 mo	Days lost: CV death/HF hosp.
CHAMPION [22] (2011)	USA	Haemodynamic (PAP)	550	61.0 years	76.0%	15 mo	HF hospitalization rate
GUIDE-HF [23] (2021)	USA/Canada	Haemodynamic (PAP)	1022	62.8 years	58.0%	12 mo	Composite HF events
Chaudhry [26] (2010)	USA	Non-inv. TM	1653	61.0 years	41.0%	6 mo	All-cause hospitalization
BEAT-HF [27] (2016)	USA	Struct. RPM	1437	73.4 years	55.2%	180 d	All-cause readmission
Brahmbhatt [28] (2024)	Canada	Struct. RPM	236	66.1 years	70.3%	6 mo	GDMT optimization score
Asch [29] (2022)	USA	Struct. RPM	500	68.7 years	44.6%	6 mo	HF hospitalization
Shara [30] (2022)	USA	Non-inv. TM	118	67.2 years	53.4%	6 mo	Composite HF events
E-INH [31] (2023)	Germany	Struct. RPM	322	66.2 years	69.3%	12 mo	All-cause mortality
Delaney [32] (2013)	USA	Non-inv. TM	148	71.0 years	52.7%	3 mo	HF hospitalization
Tompkins [33] (2010)	USA	Non-inv. TM	134	68.5 years	49.3%	6 mo	HF readmission
Pekmezaris [34] (2019)	USA	Non-inv. TM	181	63.2 years	53.0%	6 mo	All-cause hospitalization
Vuorinen [35] (2014)	Finland	Non-inv. TM	96	67.4 years	68.8%	6 mo	HF hospitalization
Seto [36] (2012)	Canada	Non-inv. TM	100	64.4 years	78.0%	6 mo	NYHA class/QoL
WISH [37] (2012)	Sweden	Non-inv. TM	168	68.0 years	62.5%	12 mo	Time to first HF event
Boyne [38] (2012)	Netherlands	Non-inv. TM	382	70.9 years	66.2%	12 mo	Composite HF events

Abbreviations: RPM = remote patient management; TM = telemonitoring; PAP = pulmonary artery pressure; GDMT = guideline-directed medical therapy; HF = heart failure; CV = cardiovascular; QoL = quality of life; F/U = follow-up; mo = months; d = days; NYHA = New York Heart Association; Non-inv. = non-invasive; Struct. = structured.

**Table 2:** Pre-specified subgroup analysis by telemedicine intervention type for all-cause mortality.

Subgroup	Trials (*n*)	Patients	RR (95% CI)	*p*-Value	I^2^ (%)	Representative Trials
Haemodynamic-guided monitoring	2	1572	0.71 (0.61–0.82)	<0.001	18%	CHAMPION [22], GUIDE-HF [23]
Structured RPM	5	4066	0.79 (0.69–0.90)	<0.001	22%	TIM-HF2 [25], E-INH [31], Brahmbhatt [28]
Non-invasive telemonitoring	9	2980	0.93 (0.84–1.03)	0.14	41%	Chaudhry [26], WISH [37], Boyne [38]
Overall (all interventions)	16	8618	0.82 (0.73–0.92)	<0.001	34%	All 16 trials [22,23,25,26,27,28,29,30,31,32,33,34,35,36,37,38]

Abbreviations: RPM = remote patient management; RR = risk ratio; CI = confidence interval; I^2^ = Cochran heterogeneity statistic.

**Table 3:** Summary of pooled outcome estimates across all 16 included RCTs.

Outcome	Studies (*n*)	Events: TM/Control	RR (95% CI)	*p*-Value	I^2^ (%)	GRADE Certainty
All-cause mortality	16	1187/1456	0.82 (0.73–0.92)	<0.001	34%	Moderate
All-cause hospitalization	14	2841/3190	0.79 (0.71–0.88)	<0.001	38%	Moderate
HF-related hospitalization	13	1604/2076	0.68 (0.59–0.78)	<0.001	29%	High
Composite (death + HF hosp.)	10	2318/2847	0.75 (0.67–0.84)	<0.001	31%	Moderate

Abbreviations: TM = telemedicine; HF = heart failure; RR = risk ratio; CI = confidence interval; I^2^ = Cochran heterogeneity statistic; GRADE = Grading of Recommendations Assessment, Development and Evaluation.

## Data Availability

No new datasets were generated for this study. All data supporting the findings of this systematic review and meta-analysis are derived from previously published studies, which are cited within the article. Additional details are available from the corresponding authors upon reasonable request.

## Supplementary Materials

The following supporting information can be downloaded at: https://www.mdpi.com/article/10.3390/jcm15103880/s1, Table S1: PRISMA checklist; Table S2: Full electronic search strategy for all databases; Table S3: GRADE Summary of Findings for primary and secondary outcomes; Figure S1: Risk of bias assessment of included randomized controlled trials using the Cochrane Risk of Bias 2 (RoB 2) tool.

## Abbreviations

The following abbreviations are used in this manuscript:

HF	Heart failure
HFrEF	Heart failure with reduced ejection fraction
HFmrEF	Heart failure with mildly reduced ejection fraction
HFpEF	Heart failure with preserved ejection fraction
RCT	Randomized controlled trial
RR	Risk ratio
CI	Confidence interval
ARR	Absolute risk reduction
NNT	Number needed to treat
RPM	Remote patient management
TM	Telemonitoring
PAP	Pulmonary artery pressure
GDMT	Guideline-directed medical therapy
NYHA	New York Heart Association
QoL	Quality of life
I^2^	Cochran’s heterogeneity statistic
τ^2^	Between-study variance
PRISMA	Preferred Reporting Items for Systematic Reviews and Meta-Analyses
GRADE	Grading of Recommendations Assessment, Development and Evaluation
CENTRAL	Cochrane Central Register of Controlled Trials
CV	Cardiovascular
