# Supplementary material for: Digital Health Strategies in Heart Failure: Effects of Telemedicine and Remote Monitoring on Clinical Outcomes—A Systematic Review and Meta-Analysis
Source: J Clin Med. 2026 May 18;15(10):3880. doi: 10.3390/jcm15103880 (PMC13207727; doi:10.3390/jcm15103880)
Supplement: Supplementary file 1 [file jcm-15-03880-s001.zip › jcm-4322650-supplementary.pdf]

Supplementary Materials

Supplementary Table S1. PRISMA Checklist.

Section and Topic	Item #	Checklist item	Location where item is reported
**TITLE**			
Title	1	Identify the report as a systematic review.	Title page
**ABSTRACT**			
Abstract	2	See the PRISMA 2020 for Abstracts checklist.	Abstract (p.1–2)
**INTRODUCTION**			
Rationale	3	Describe the rationale for the review in the context of existing knowledge.	Introduction (p.2–3)
Objectives	4	Provide an explicit statement of the objective(s) or question(s) the review addresses.	End of Introduction (last paragraph, p.3)
**METHODS**			
Eligibility criteria	5	Specify the inclusion and exclusion criteria for the review and how studies were grouped for the syntheses.	Section 2.2
Information sources	6	Specify all databases, registers, websites, organisations, reference lists and other sources searched or consulted to identify studies. Specify the date when each source was last searched or consulted.	Section 2.3
Search strategy	7	Present the full search strategies for all databases, registers and websites, including any filters and limits used.	Supplementary Table S2
Selection process	8	Specify the methods used to decide whether a study met the inclusion criteria of the review, including how many reviewers screened each record and each report retrieved, whether they worked independently, and if applicable, details of automation tools used in the process.	Section 2.4
Data collection process	9	Specify the methods used to collect data from reports, including how many reviewers collected data from each report, whether they worked independently, any processes for obtaining or confirming data from study investigators, and if applicable, details of automation tools used in the process.	Section 2.5
Data items	10a	List and define all outcomes for which data were sought. Specify whether all results that were compatible with each outcome domain in each study were sought (e.g. for all measures, time points, analyses), and if not, the methods used to decide which results to collect.	Section 2.8 (Outcomes)
	10b	List and define all other variables for which data were sought (e.g. participant and intervention characteristics, funding sources). Describe any assumptions made about any missing or unclear information.	Section 2.5
Study risk of bias assessment	11	Specify the methods used to assess risk of bias in the included studies, including details of the tool(s) used, how many reviewers assessed each study and whether they worked independently, and if applicable, details of automation tools used in the process.	Section 2.6; Supplementary Figure S1

Section and Topic	Item #	Checklist item	Location where item is reported
Effect measures	12	Specify for each outcome the effect measure(s) (e.g. risk ratio, mean difference) used in the synthesis or presentation of results.	Section 2.9
Synthesis methods	13a	Describe the processes used to decide which studies were eligible for each synthesis (e.g. tabulating the study intervention characteristics and comparing against the planned groups for each synthesis (item #5)).	Section 2.2 & 2.10
	13b	Describe any methods required to prepare the data for presentation or synthesis, such as handling of missing summary statistics, or data conversions.	Section 2.5 & 2.9
	13c	Describe any methods used to tabulate or visually display results of individual studies and syntheses.	Section 2.9; Figures 1–2; Tables 1–3
	13d	Describe any methods used to synthesize results and provide a rationale for the choice(s). If meta-analysis was performed, describe the model(s), method(s) to identify the presence and extent of statistical heterogeneity, and software package(s) used.	Section 2.9
	13e	Describe any methods used to explore possible causes of heterogeneity among study results (e.g. subgroup analysis, meta-regression).	Section 2.10
	13f	Describe any sensitivity analyses conducted to assess robustness of the synthesized results.	Section 2.11
Reporting bias assessment	14	Describe any methods used to assess risk of bias due to missing results in a synthesis (arising from reporting biases).	Section 2.12
Certainty assessment	15	Describe any methods used to assess certainty (or confidence) in the body of evidence for an outcome.	Section 2.7
**RESULTS**			
Study selection	16a	Describe the results of the search and selection process, from the number of records identified in the search to the number of studies included in the review, ideally using a flow diagram.	Section 3.1; Figure 1
	16b	Cite studies that might appear to meet the inclusion criteria, but which were excluded, and explain why they were excluded.	Section 3.1
Study characteristics	17	Cite each included study and present its characteristics.	Section 3.2; Table 1
Risk of bias in studies	18	Present assessments of risk of bias for each included study.	Section 3.3; Figure S1
Results of individual studies	19	For all outcomes, present, for each study: (a) summary statistics for each group (where appropriate) and (b) an effect estimate and its precision (e.g. confidence/credible interval), ideally using structured tables or plots.	Section 3.4; Figure 2
Results of syntheses	20a	For each synthesis, briefly summarise the characteristics and risk of bias among contributing studies.	Section 3.2 & 3.5
	20b	Present results of all statistical syntheses conducted. If meta-analysis was done, present for each the summary estimate and its precision (e.g. confidence/credible interval)	Sections 3.4–3.6; Tables 2–3

Section and Topic	Item #	Checklist item	Location where item is reported
		and measures of statistical heterogeneity. If comparing groups, describe the direction of the effect.	
	20c	Present results of all investigations of possible causes of heterogeneity among study results.	Section 3.5
	20d	Present results of all sensitivity analyses conducted to assess the robustness of the synthesized results.	Section 3.7
Reporting biases	21	Present assessments of risk of bias due to missing results (arising from reporting biases) for each synthesis assessed.	Section 3.8
Certainty of evidence	22	Present assessments of certainty (or confidence) in the body of evidence for each outcome assessed.	Section 3.9; Table S3
**DISCUSSION**			
Discussion	23a	Provide a general interpretation of the results in the context of other evidence.	Section 4.1–4.3
	23b	Discuss any limitations of the evidence included in the review.	Section 4.6
	23c	Discuss any limitations of the review processes used.	Section 4.6
	23d	Discuss implications of the results for practice, policy, and future research.	Section 4.7
**OTHER INFORMATION**			
Registration and protocol	24a	Provide registration information for the review, including register name and registration number, or state that the review was not registered.	Section 2.1
	24b	Indicate where the review protocol can be accessed, or state that a protocol was not prepared.	Section 2.1
	24c	Describe and explain any amendments to information provided at registration or in the protocol.	Not applicable
Support	25	Describe sources of financial or non-financial support for the review, and the role of the funders or sponsors in the review.	Funding section
Competing interests	26	Declare any competing interests of review authors.	Conflicts of Interest
Availability of data, code and other materials	27	Report which of the following are publicly available and where they can be found: template data collection forms; data extracted from included studies; data used for all analyses; analytic code; any other materials used in the review.	Data Availability Statement

Supplementary Table S2. Full electronic search strategy for all databases.

Database	Search Strategy
PubMed/MEDLINE	("heart failure"[MeSH Terms] OR "heart failure"[Title/Abstract] OR "cardiac failure"[Title/Abstract]) AND ("telemedicine"[MeSH Terms] OR "telemedicine"[Title/Abstract] OR "telemonitoring"[Title/Abstract] OR "remote monitoring"[Title/Abstract] OR "remote patient monitoring"[Title/Abstract] OR "remote patient management"[Title/Abstract] OR "digital

Database	Search Strategy
	health"[Title/Abstract]) AND ("randomized controlled trial"[Publication Type] OR "randomized controlled trial"[Title/Abstract] OR "randomised controlled trial"[Title/Abstract] OR "RCT"[Title/Abstract])
Embase	('heart failure'/exp OR 'heart failure':ti,ab OR 'cardiac failure':ti,ab) AND ('telemedicine'/exp OR 'telemedicine':ti,ab OR 'telemonitoring':ti,ab OR 'remote monitoring':ti,ab OR 'remote patient monitoring':ti,ab OR 'remote patient management':ti,ab OR 'digital health':ti,ab) AND ('randomized controlled trial'/exp OR 'randomized controlled trial':ti,ab OR 'randomised controlled trial':ti,ab OR 'RCT':ti,ab)
Cochrane CENTRAL	("heart failure" OR "cardiac failure") AND ("telemedicine" OR "telemonitoring" OR "remote monitoring" OR "remote patient monitoring" OR "remote patient management" OR "digital health") AND ("randomized controlled trial" OR "RCT")

Searches were conducted from database inception to 15 March 2026. No language restrictions were applied during the initial search; however, only studies published in English were included in the final analysis.

Supplementary Table S3. GRADE Summary of Findings for primary and secondary outcomes.

Outcome	No. of Studies (Participants)	Relative Effect (RR, 95% CI)	Absolute Effect	Certainty of Evidence (GRADE)	Comments
All-cause mortality	16 RCTs (n = 12,847)	0.82 (0.73–0.92)	41 fewer per 1000 (from 18 to 61 fewer)	⊕⊕⊕◯ Moderate	Downgraded for inconsistency (I^2 = 34%); mechanistic heterogeneity across interventions
All-cause hospitalization	14 RCTs (n ≈ 12,000)	0.79 (0.71–0.88)	63 fewer per 1000 (from 36 to 87 fewer)	⊕⊕⊕◯ Moderate	Downgraded for inconsistency (I^2 = 38%); composite endpoint variability
HF-related hospitalization	13 RCTs (n ≈ 11,000)	0.68 (0.59–0.78)	96 fewer per 1000 (from 66 to 123 fewer)	⊕⊕⊕⊕ High	Strong, consistent effect with low heterogeneity (I^2 = 29%)

Outcome	No. of Studies (Participants)	Relative Effect (RR, 95% CI)	Absolute Effect	Certainty of Evidence (GRADE)	Comments
Composite (death + HF hospitalization)	10 RCTs (n ≈ 10,000)	0.75 (0.67–0.84)	79 fewer per 1000 (from 50 to 104 fewer)	⊕⊕⊕◯ Moderate	Downgraded for heterogeneity in composite endpoint definitions

CI: confidence interval; RR: risk ratio; HF: heart failure; GRADE: Grading of Recommendations Assessment, Development and Evaluation.

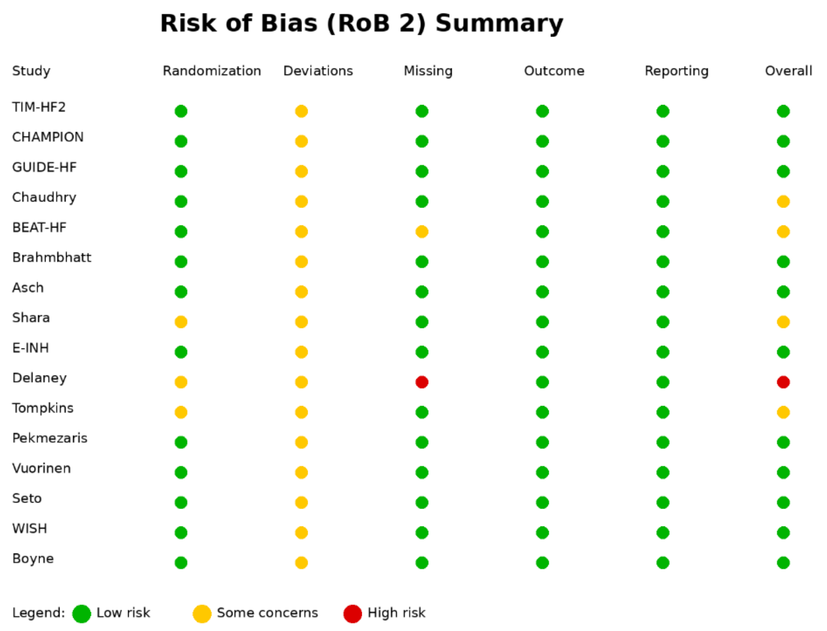

Figure S1. Risk of bias assessment of included randomized controlled trials using the Cochrane Risk of Bias 2 (RoB 2) tool. Each domain is rated as low risk (green), some concerns (yellow), or high risk (red).
